# Detection of Cartilage Oligomeric Matrix Protein Using a Quartz Crystal Microbalance

**DOI:** 10.3390/s101211633

**Published:** 2010-12-20

**Authors:** Shih-Han Wang, Chi-Yen Shen, Ting-Chan Weng, Pin-Hsuan Lin, Jia-Jyun Yang, I-Fen Chen, Shyh-Ming Kuo, Shwu-Jen Chang, Yuan-Kun Tu, Yu-Hsien Kao, Chih-Hsin Hung

**Affiliations:** 1 Department of Chemical Engineering, I-Shou University, No. 1, Sec. 1, Syuecheng Rd., Dashu Township, Kaohsiung County 840, Taiwan; E-Mails: shwang@isu.edu.tw (S.-H.W.); sophia19870306@hotmail.com (P.-H.L); lou790516@hotmail.com (J.-J.Y.); 2 Department of Electrical Engineering, I-Shou University, Taiwan; E-Mails: cyshen@isu.edu.tw (C.-Y.S.); q70049@hotmail.com (T.-C.W.); 3 Department of Biomedical Engineering, I-Shou University, No.8, Yi-Da Road, Jiau-shu Tsuen, Yan-chau Shiang, Kaohsiung County, Taiwan; E-Mails: ifen@isu.edu.tw (I.-F.C.); smkuo@isu.edu.tw (S.-M.K); sjchang@isu.edu.tw (S.-J.S.); 4 Department of Orthopaedic Surgery, E-Da Hospital; No.1, Yi-Da Road, Jiau-shu Tsuen, Yan-chau Shiang, Kaohsiung County, Taiwan; E-Mails: ed100130@edah.org.tw (Y.-K.T.); ed102523@edah.org.tw (Y.-H.K.)

**Keywords:** immunosensor, quartz crystal microbalance (QCM), cartilage oligomeric matrix protein (COMP), urinary biomarker

## Abstract

Current methods for diagnosing early stage osteoarthritis (OA) based on the magnetic resonance imaging and enzyme-linked immunosorbent assay methods are specific, but require specialized laboratory facilities and highly trained personal to obtain a definitive result. In this work, a user friendly and non-invasive quartz crystal microbalance (QCM) immunosensor method has been developed to detect Cartilage Oligomeric Matrix Protein (COMP) for early stage OA diagnosis. This QCM immunosensor was fabricated to immobilize COMP antibodies utilizing the self-assembled monolayer technique. The surface properties of the immunosensor were characterized by its FTIR and electrochemical impedance spectra (EIS). The feasibility study was based on urine samples obtained from 41 volunteers. Experiments were carried out in a flow system and the reproducibility of the electrodes was evaluated by the impedance measured by EIS. Its potential dynamically monitored the immunoreaction processes and could increase the efficiency and sensitivity of COMP detection in laboratory-cultured preparations and clinical samples. The frequency responses of the QCM immunosensor changed from 6 kHz when testing 50 ng/mL COMP concentration. The linear regression equation of frequency shift and COMP concentration was determined as: y = 0.0872 x + 1.2138 (R^2^ = 0.9957). The COMP in urine was also determined by both QCM and EIS for comparison. A highly sensitive, user friendly and cost effective analytical method for the early stage OA diagnosis has thus been successfully developed.

## 1. Introduction

Osteoarthritis (OA), the impairment of joint disease, is a progressive destruction of articular cartilage and subchondral bone, accompanying by synovial change. OA is a prevalent cause of pain and disability in a considerable proportion of the aging population. No method or drug has been proven to stop disease progression or make cartilage rejuvenate. There is no proper detection method to diagnose the initial cartilage degradation of OA and to determine exact therapies. Planar radiographs were used in detecting joint space width, but the cartilage destruction could only be determined from radiographs when significant cartilage degradation has occurred. Therefore, early diagnostics of OA symptoms by biochemical methods or sensor systems is an urgent necessity. A delayed gadolinium-enhanced magnetic resonance imaging of cartilage (dGEMRIC) method was designed to examine glycosaminoglycan changes in articular cartilage during the development of OA. However, dGEMRIC is not available in most clinic facilities, it tests are lenghty and patients are also exposed to high radiation doses when cartilage tissue is measured by this method. On the other hand, biological markers might provide sufficient information to reveal dynamic changes of the cartilage. Several studies have shown that serum levels of cartilage oligomeric matrix protein (COMP), which is abundant in OA cartilage, are a sensitive marker for cartilage degradation detection and thus a potential prognostic marker providing important information on metabolic changes occurring in the cartilage matrix in joint diseases [1–4]. The COMP levels in serum can be detected by the enzyme-linked immunosorbent assay (ELISA) method, which is a typical biochemical assay used mainly in immunology to detect the presence of COMP in a sample [5], but ELISA immunoassays are in general costly, requiring complex procedures using expensive laboratory equipment, long analysis times and the participation of highly skilled operators.

Considerable efforts have been directed towards the development of simple biosensors for the detection of viruses [6–11]. Biosensors can detect interactions between viral antigens, bacterium, protein particles and DNA by specific antibodies and can be classified according to the type of transducer used in the device [8,9]. Piezoelectric sensors, such as the quartz crystal microbalance (QCM), are the potential candidates for biosensors. An electrical field, applied to the QCM, produces mechanical stresses that induce an acoustic wave to travel in a direction perpendicular to the surfaces of the crystal. Biological compounds such as antibodies are capable of binding to terminal active functional groups (*i.e.*, COOH, OH and NH_2_) of self-assembled monolayers (SAM) and immunocapture antigens such as COMP or other targets. The QCM can consequently detect mass changes due to these molecular interactions on the surface of the QCM.

Sauerbrey first described the relationship between frequency shift and mass change on the crystal surface in air [12]. The frequency response of the QCM is also dependent on both the density and viscosity of the solution as a liquid passes over the QCM crystal surface [13]. The QCM device is convenient to use and it rapidly detects in real-time the responses of antigen–antibody interactions on the surface of device [14,15]. Therefore, the low cost and easy operated QCM device has been applied in various biotechnology fields, such as clinical diagnosis [16–18] and environmental monitoring [19].

Most biochemical diagnoses of cartilage degradation use synovial fluid from invasive operations at diseased sites or in serum. There is very little literature in which the COMP concentration in urine of OA patients has been defined. In order to develop an easy to perform and homecare system for monitoring the cartilage degradation, a non-invasive simple QCM-based sensor was developed in this research. The efficiency and sensitivity of the sensor were also evaluated to further enhance its practicability in early OA diagnosis

## 2. Experimental Section

### 2.1. Materials

COMP Human, Mouse Monoclonal Antibody, Clone:16F12 was purchased from BioVendor (Candler, NC, USA) and Recombinant Human COMP (>90%) was purchased from R&D Systems (Minneapolis, MN, USA). N’-(3-dimethylaminopropyl)-3-ethyl carbodiimide hydrochloride (EDC, 99%), medium for preparing phosphate buffer saline (PBS, 137 mM NaCl, 2.7 mM KCl, 10 mM Na_2_HPO_4_, 2 mM KH_2_PO_4_, pH 7.4) and bovine serum albumin were purchased from Sigma (St. Louis, MO, USA). Thioctic Acid (TA, >98%) was obtained from ACROS (USA). The electrolyte potassium ferricyanide (K_3_[Fe(CN)_6_], SHOWA), potassium ferrocyanid (K_4_[Fe(CN)_6_], SHOWA) and potassium chloride (KCl, Sigma) were analytical grade. Doubly distilled water was used throughout the experiments. The feasibility study was carried out using urine samples from 41 persons including 14 males and 27 females, collected from healthy personnel and hospital OA patients. The samples were provided by E-Da hospital, Kaohsiung, Taiwan, and analyzed without further treatment.

### 2.2. Sensor Surface Modification

The QCM sensor (Taitien Co., Ltd, Taiwan), coupled inside a flow injection system, was a 10 MHz quartz crystal with a 3.8 mm diameter gold electrode. Each of the gold electrodes was pretreated by electrochemical cleaning in 0.5 M H_2_SO_4_ solution using cyclic voltammetry at a scan rate of 100 mV/s for five cycles and then washed in de-ionized water and dried with a light stream of nitrogen gas. The pretreated gold electrode was immersed in the 2.5 mM thioctic acid (TA) alcohol solution at room temperature for 24 h in the darkroom. Afterwards, it was rinsed thoroughly with ethanol and dried with nitrogen gas and stock at room temperature for further used.

### 2.3. Immobilization of COMP Monoclonal Antibody

**Scheme 1.**
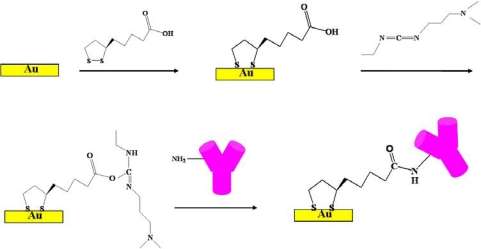
Schematic diagram of the immobilization procedure.

The coupling agent, 0.2 M 1-ethyl-3-(3-dimethylaminopropyl) carbodiimide hydrochloride (EDC) was used to activated the prepared TA monolayer for 3 hr at room temperature and then rinsed with ethanol and dried as aforementioned. Then 20 ìL of COMP monoclonal antibody (0.01 mg mL^−1^) in PBS solution was placed on the electrode to conjugate at 4 °C for 12 h and then rinsed by PBS. Afterwards, the electrode was blocking by 5% bovine serum albumin (BSA) for 1.5 h. Finally, the electrode was rinsed with PBS and then dried by nitrogen gas. Scheme 1 illustrates the schematic diagram of the COMP antibody immobilization procedure.

The chemical structure of the modified electrodes was characterized by Fourier Transfer Infrared Spectrometry (FTIR, Nicolet 5700) and the impedances of the electrodes were analyzed by electrochemical impedance spectrometry (EIS, CHI 614 B). The EIS analysis was accomplished in a three-electrode mode system wherein the modified gold electrode, a screen-printed carbon electrode and an external Ag/AgCl electrode were working, counter and reference electrode, respectively.

### 2.4. Measurement

**Figure 1.**
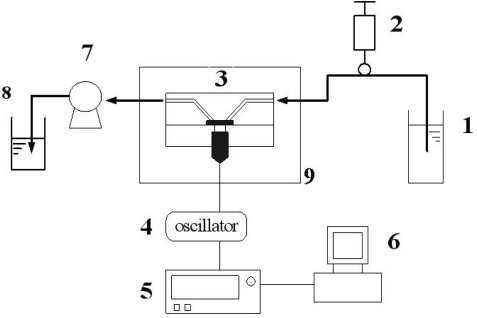
A schematic diagram of the apparatus. 1: liquid tank; 2: inject port; 3: flow-through cell; 4: oscillator; 5: frequency counter; 6: computer; 7: pump; 8: waste; 9: chamber.

Figure 1 presents a schematic diagram of the apparatus used in this work. A frequency counter collected the output signal of the oscillator. The prepared QCM immunsensor was mounted on one side of the detection vessel. PBS solution with pH 7.4 was prepared to be an assay buffer solution and was injected into the vessel to stabilize the equipment. After stabilization of the resonance frequency of QCM, the COMP solution (4 mL of 0 ng/mL to 80 ng/mL) or the urine sample (4 mL) was then introduced into the detection vessel. The frequency counter recorded the frequency shift when the immunoreactions proceeded until equilibrium was reached 25 min in order to avoid the response induced by non-specific adsorption. The frequency shifts in all experiments were calculated on the average responses of the immunoreactions with corresponding standard deviations of triplicate measurements. The impedance of electrodes in different sample concentration was analyzed by EIS at 30 °C after immersion the electrode in 20 μL sample solution for 5 min and following by PBS rinse. The EIS analysis was accomplished with three-electrode mode in the PBS solution with 5 mM Fe(CN)_6_^3−/4−^ and KCl.

## 3. Results and Discussion

### 3.1. Characterization of the Modified Electrode

**Figure 2.**
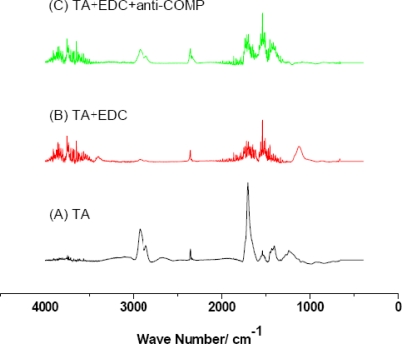
Surface characterization of the electrodes. (A–C) FTIR spectrums of the modified gold electrodes in different stapes (A) thiotic acid; (B) EDC; (C) COMP monoclonal antibody.

In order to determine the chemical structure of the modified gold electrode, each electrode sample was characterized by FTIR. Figure 2(a) shows the FTIR spectra of modified electrodes at different density stages.

For the thiotic acid modified electrode the C=O and C-H functional groups appeared at 1,700∼1,730 cm^−1^ and 2,900 cm^−1^, respectively. It suggested that the thiotic acid was successfully modified onto the electrode surface. In Figure 2(b), the C-N vibration peak at 1,150 cm^−1^ and the enhanced N-H vibration peaks at 3,400 cm^−1^ implied that the carboxylic acid group was activated by EDC. In Figure 2(c), the C-H and N-H vibration peaks suggest that the anti-COMP layer was successfully immobilized onto the electrode surface.

**Figure 3.**
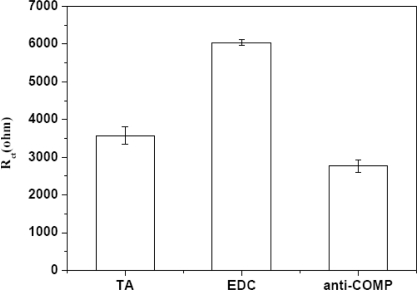
Impedance of the gold electrode immobilized by COMP monoclonal antibody and the electrode after BSA treatment (nine different electrodes).

The reproducibility of the modified gold electrodes was evaluated by examining the impedance for each electrode. The impedance of the COMP antibody immobilized electrode and the BSA treated electrode were evaluated by EIS at 30 °C. All electrochemical measurements were performed in a three-electrode electrochemical cell. A screen printed carbon and a screen printed Ag/AgCl electrodes were used as the counter and reference electrode, respectively. A QCM was introduced as the working electrode. A thioctic acid monolayer was formed by the SAMs technique, and then the functional group of TA monolayer was activated by EDC. Finally anti-COMP and BSA were immobilized on modified electrode area successfully. Impedance spectroscopy (EIS) studies demonstrated that the formation of antibody–antigen complexes increased the electron-transfer resistance (R_ct_) of Fe(CN)_6_^3−/4−^ redox pair at the BSA/anti-COMP/EDC/TA/gold electrode. Figure 3 represents the EIS spectra for different electrodes, and the average impedances for the COMP antibody modified electrode was 2,766 Ω. The relatively low deviation implied that the high reproducibility and high reliability of the electrodes utilizing this SAM immobilization technique.

### 3.2. QCM Immunosensor for Detecting COMP

**Figure 4.**
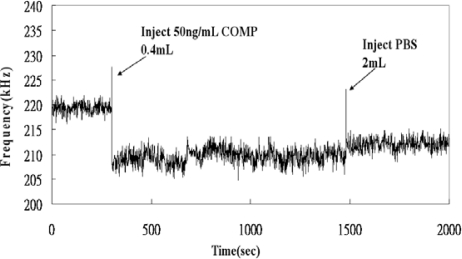
Frequency responses of the developed QCM immunosensor.

The binding capacity of the proposed QCM immunosensor was examined by detecting various concentrations of COMP. Figure 4 showed the typical frequency responses monitored by the QCM immunosensor for COMP detection at 26 °C. When the QCM immunosensor detected COMP concentration at 50 ng/mL, the frequency response quickly shifted downward from 219 to 213 kHz. The temperature of the sample fluids should be strictly controlled because it can strongly affect the immunological reactions; hence a fluid temperature controller was also incorporated in our QCM device.

We compared four different fluid temperatures to determine the optimum working conditions for the immunological reactions. At 25 °C and 26 °C the reactions were most effective, and there was no distinct difference between 25 °C and 26 °C. Compared to the ELISA method that requires more than 30 min identification time, the QCM immunosensor could identify COMP in a few seconds. Besides the time advantage, the complicated procedures and expensive experimental materials of the ELISA assay make it difficult to become a homecare system.

### 3.3. High Correlation Between of COMP Concentration and Frequency

**Figure 5.**
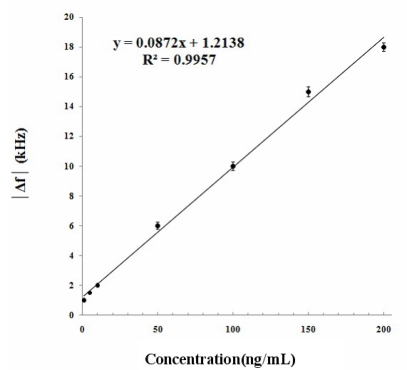
Calibration curve: Relationship between COMP concentration and observed frequency shift at 26 °C.

According to the data from the commercial ELISA kit (BioVendor) when detecting COMP, the calibration standard curve of human COMP concentration was established according to the kit protocol using the concentration range from 4–128 ng/mL. The QCM sensor device for COMP detection should also have high response and sensitivities to reflect the real concentrations of COMP in detecting sample. In this study, a linear relationship between COMP concentration and frequency shift at 26 °C was shown in Figure 5.

The linear regression equation is y = 0.0872 x + 1.2138 (R^2^ = 0.9957), where y is absolute value of frequency shift and x is COMP concentration in ng/mL. The frequency shifts were observed in the range of 1–200 ng/mL, which showed the QCM sensors have higher sensitivity and faster response than the ELISA method in the real time detection, thus allowing the possibility of estimating COMP concentrations in unknown samples.

### 3.4. EIS Analysis of COMP Concentration

**Figure 6.**
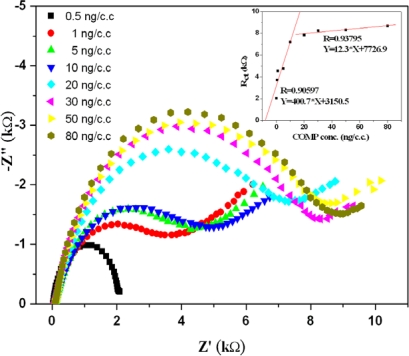
The Nyquist plot of the gold electrodes in different COMP aqueous solutions.

Since the impedance of the electrode was significantly altered by the electrode surface condition, we exploited the EIS technique to detect the COMP concentration. The modified electrodes were immersed in 20 μL of solution with the desired COMP concentration for 5 min and then rinsed with PBS. Due to the different amounts of COMP adsorption, the impedance of the electrodes changed with the COMP concentrations as shown in Figure 6. Impedance spectroscopy (EIS) studies demonstrated that the formation of antibody–antigen complexes increased the electron-transfer resistance (R_ct_) of Fe(CN)_6_^3−/4−^ redox pair at the BSA/anti-COMP/EDC/TA/gold electrode. Because of the relatively large surface area for EIS analysis, the sensitivity of the electrode was higher at relatively low COMP concentrations. Nevertheless, the operating procedure of EIS is much simpler than the ELISA method and it could therefore be applied as a homecare system.

### 3.5. Monitoring of COMP Binding in Urine

**Figure 7.**
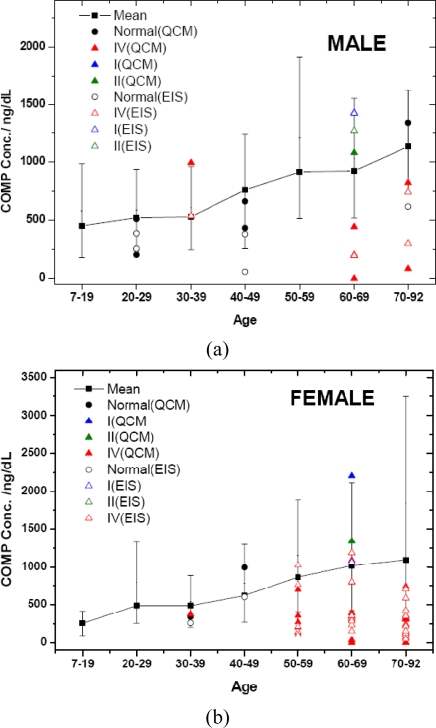
Detection of the urine COMP level of 41 volunteers’ urine samples by QCM sensor (a) and ELISA kit [20] (b). I, II, IV: Osteoarthritis Research Society International (OARSI) histological grade in the progression of osteoarthritis.

This QCM based sensor needs to be further evaluated for its rapid and sensitive detection of COMP in practical clinical specimens of OA. We collected 41 urine samples including patients with OA and normal persons to compare the measurement results of the QCM and electrochemical immunosensor. According to the ELISA kit obtained from BioVendor [20], the COPM concentration in serum was relatively higher than that in urine. COMP concentrations of 41 urine specimens measured by the EIS assay and the QCM sensor are shown in Figure 7. The high, low and mean data was the serum COMP concentrations from 246 unselected blood donors assayed with the Human COMP ELISA kit obtained from BioVendor [20].

The trends of the results obtained by QCM and EIS were similar. Comparisons of ELISA data, QCM results and the reference values of COMP concentrations [20], showed the COMP concentration measured by QCM and EIS sensor showed results consistent with the traditional ELISA detection. The urine COMP level in patients with grade 1–2 of OA presented higher concentrations than the mean level in normal persons. However, the urine COMP level in patients with grade 4 OA was very low. It was because that cartilage erosion in a patient with grade 4 of OA is very serious, therefore the cartilage was almost decayed and no more COMP was released to be detected. The developed QCM immunosensor provides a rapid and sensitive measure for detecting the presence of COMP, and therefore can be applied to early diagnosis of OA.

## 4. Conclusions

Clinical diagnosis of OA is difficult, especially in the early stages of cartilage degradation. Most methods for OA diagnosis involve invasive collection of specimens or radiophotography that could expose patients on the radiation. Most patients that have been defined as OA have cartilage erosion with fast degradation of cartilage, so early OA detection is important for early cartilage protection or medical treatments. A detection system and device for easy operation, like the device for detecting blood glucose, should be helpful for homecare. This study used the well known COMP antibody biomarker immobilized on a QCM sensor and established its stable detection properties at room temperature. From the data of this study, such a QCM sensor showed the same sensitivity as EIS, and the values of COMP of volunteers also reflect the grades of cartilage degradation determined by clinical diagnosis. In addition, the analytical procedures of this QCM immunosensor are direct and simple in real time without multiple labeling and separation steps. The experimental results suggested that a highly sensitive and user friendly QCM sensor has been successfully developed for early stage OA diagnosis.
